# Plant health: feedback effect of root exudates-rhizobiome interactions

**DOI:** 10.1007/s00253-018-9556-6

**Published:** 2018-12-20

**Authors:** Oluwaseyi Samuel Olanrewaju, Ayansina Segun Ayangbenro, Bernard R. Glick, Olubukola Oluranti Babalola

**Affiliations:** 1 Food Security and Safety Niche Area, Faculty of Natural and Agricultural Sciences, North-West University, Mmabatho, 2735 South Africa; 2 Department of Biology, University of Waterloo, Waterloo, ON N2L 3G1 Canada

**Keywords:** MAMPs, Plant-microbe interaction, PEPs, Quorum sensing, Rhizobiome, Root exudates

## Abstract

The well-being of the microbial community that densely populates the rhizosphere is aided by a plant’s root exudates. Maintaining a plant’s health is a key factor in its continued existence. As minute as rhizospheric microbes are, their importance in plant growth cannot be overemphasized. They depend on plants for nutrients and other necessary requirements. The relationship between the rhizosphere-microbiome (rhizobiome) and plant hosts can be beneficial, non-effectual, or pathogenic depending on the microbes and the plant involved. This relationship, to a large extent, determines the fate of the host plant’s survival. Modern molecular techniques have been used to unravel rhizobiome species’ composition, but the interplay between the rhizobiome root exudates and other factors in the maintenance of a healthy plant have not as yet been thoroughly investigated. Many functional proteins are activated in plants upon contact with external factors. These proteins may elicit growth promoting or growth suppressing responses from the plants. To optimize the growth and productivity of host plants, rhizobiome microbial diversity and modulatory techniques need to be clearly understood for improved plant health.

## Introduction

There is a growing demand for increased crop yields and sustainable agriculture as a result of the ever-increasing world population. This puts ever-increasing pressure on the soil to facilitate greater plant productivity. Soil contains both an enormous and highly diverse microbial community, and it is precisely this massive collection of microorganisms, living in intimate association with plants, either in the rhizosphere, or within plant tissues, or as epiphytes attached to aboveground plant tissue, that has a critical impact on plant growth and development (Vorholt 2012). These microbes may be beneficial, or harmful to plant growth and development, or they may have no discernible impact on plants whatsoever. Plants and the root microbiome, which is comprised of the communities existing in the plant root ecosystem, interact for disease suppression, increased nutrition, and better growth promotion (Lakshmanan et al. 2014).

Microbes in the rhizosphere can help plants to grow and function more effectively by increasing plant pathogen resistance, retain more water, take up and utilize more nutrients and, in general, increase their growth. Plants, for their part, exude a range of carbon metabolites that can act as a food and energy source for use by the microbes. As discussed below, some microbes are pathogenic and hence negatively affect the growth and development of plants. The plant root microbiome is important, its multifariousness is determined by the soil’s physicochemical properties and the type of host plants (Yuan et al. 2015). The relationship between plants and rhizospheric microbes has been comprehensively studied for biocontrol, plant growth promoting, and biogeochemical cycling activities, all of which are essential for the good health of the plants. This has necessitated the need to better understand the rhizosphere microbial diversity so that plant beneficial microbes may be optimally utilized in terms of plant growth promotion. The plant root system is the main medium of exudation into the rhizosphere. Moreover, the growth of plant root systems is controlled by different soil physicochemical properties, properties that are in turn partly modified and influenced by roots themselves. Many factors such as the effect on the rhizosphere and soil organic matter need to be considered in root exudate-rhizobiome interactions. However, only rhizobiome-plant health effects will be discussed in this review.

Recent interests in subsurface ecology research and the advent of next-generation sequencing (NGS) techniques have helped to elaborate a number of important interactions that occur in the rhizosphere, many of which are facilitated by root-secreted photo-assimilates. Root exudates indirectly regulate the impacts of biotic and abiotic processes by determining the microbial community, affecting the control against herbivores, and changing the physicochemical properties of the rhizospheric soil for a plant’s benefit (Bakker et al. 2018; Lladó et al. 2018). Photosynthates are comprised mainly of carbon compounds, electrons, protons, water, and inorganic ions, which all enter the rhizosphere as root exudates. Aside from the photosynthates, phytosiderophores and polysaccharides form a large part of the rhizospheric deposits. The former aids in nutrient acquisition, while the latter, in connection with rhizosphere microbes, forms a mucigel that provides protection and the free flow of movement in the rhizosphere for symbiotic microbes. These interactions can also be aided by exudates acting as signal molecules thereby directly mediating plant-microbe interactions, plant-plant interactions, and microbe-microbe interactions in the rhizosphere.

### Rhizobiome, root exudates, and plant health: the story so far

#### Rhizobiome

The term microbiome has been defined as the total microbes in a community (Orozco-Mosqueda et al. 2018). Bearing this in mind, we define the rhizobiome, taken from rhizosphere microbiome, as all of the microbes present in the plant rhizosphere.

Berendsen et al. (2012) referred to the complex community in a root microbiome as a plant’s second genome consisting of the totality of the rhizosphere community’s (including the microbes and genetic elements present) interactions in relation to plant health. Rhizobiome functioning in plant growth promotion has been recognized, but the different interactions involved have not been studied in any detail due to the lack of availability of the required tools and techniques. However, some achievements in the understanding of the root microbiome through different techniques have been reported. For example, the advent of advanced sequencing technologies has helped in the studies of the rhizobiome (Schlaeppi and Bulgarelli 2015; Turner et al. 2013). These techniques, combined with others such as metagenomics, proteomics, metabolomics, transcriptomics, and metatranscriptomics will ultimately prove to be helpful in rhizospheric microbial diversity studies, as well as helping to elucidate the relationship between the rhizobiome and the plant, including how this relationship affects a plant’s health status.

The rhizobiome may consist of either beneficial or non-beneficial microbes. According to Holden et al. (2009), microsites present on plant hosts may allow for inter-kingdom jumps by human pathogenic bacteria. Plant genotype, species, and the soil constituents largely determine a plant’s rhizobiome (Bakker et al. 2012), so that the microbes become specific to their biome. This is evident in the stimulation of *Bacillus subtilis* by malic acid present in the rhizobiome (Lakshmanan et al. 2014; Mendes et al. 2013). The rhizobiome can influence the plant community, leading to mutualistic coexistence of competitors in the same environment (Liu et al. 2015). It can either be a positive association, involving host symbiosis, or a negative one, involving pathogens and predators, or neutral (Bever et al. 2012). Whether positively or negatively, the rhizobiome affects plant growth and stress tolerance and its importance is gaining more attention (Mendes et al. 2013).

### Root exudate-rhizobiome relationship

The first reported study of the root-microbe relationship was done by Foster and Rovira (1976) who examined ultrathin sections of the wheat rhizobiome, using transmission electron microscopy. Immature roots were observed to be sparingly colonized by microbes, while the opposite was observed in the rhizobiome, cortical cells, and their cell walls. Furthermore, rhizospheric bacteria were significantly different from those in the bulk soil, both in number and type. There were size differences as well with ~ 80% of the bacteria with a diameter greater than 0.3 μm compared to ~ 37% in the bulk soil. Outside of the rhizoplane, the bacteria were found in distinctly isolated colonies colligated with organic debris with larger colonies associated with cell wall remnants. Although the total biomass of fungi and protozoa, which are both typically much larger than bacteria, may be similar to bacteria, their frequency in the rhizobiome is much lower than that of bacteria (Zhou et al. 2016).

Many processes in the rhizobiome do not occur passively without being acted upon by an external body. It may be the case that there is an intermediary serving as a connector to link up the mediators of the processes, or there might be a signal that determines the beginning or end of a process. However, most interactions need a link that connects the mediators together. This is where root exudates come into play. They may repel or attract (recruit) microbes to the rhizobiome, linking various interactions occurring in the rhizobiome, exerting a significant effect on the general health of the plants even though at least a portion of the exudates have traditionally been considered to be plant wastes (Bais et al. 2006; Peter 2011). In this regard, while knowledge of the biochemistry, biology, and genetics of root development has significantly increased in the last few years, the processes involved in rhizobiome interactions as a consequence of exudate secretion are not yet well understood (Hayat et al. 2017).

Roots provide plants with mechanical support and a means for the uptake of nutrients and water. In addition, the rhizosphere is a hotspot for soil microbes (Kuzyakov and Blagodatskaya 2015) because the compounds secreted by the roots are important signals for microbes as they can either attract or repel microbes to the plant (Lakshmanan et al. 2014) suggesting that root exudates regulate the interaction between the roots and soil microbes (Mommer et al. 2016). Shared components of signal pathways in the rhizosphere induce a high level of plant-microbe, microbe-microbe, and plant-plant interaction thereby regulating and inducing responses in the rhizosphere.

Root exudates and other rhizodeposition secreted by plants help to determine the microbiota present in the rhizobiome of plants (Moe 2013). Not all root exudates are directly involved in plant nutrition and growth. Some act as signal molecules mediating interactions in the rhizobiome. Among the exudates are sugars including monosaccharides (such as fructose, mannose, and glucose), disaccharides (maltose), five carbon sugars (arabinose), and oligosaccharides; amino acids including aspartate, asparagine, glutamine, arginine, and cysteine; organic acids such as ascorbic, acetic, benzoic, ferulic, and malic acids; phenolic compounds such as coumarin; and some high-molecular-weight compounds such as flavonoids, enzymes, fatty acids, auxin, gibberellin, nucleotides, tannins, steroids, terpenoids, alkaloids, polyacetylenes, and vitamins (Gunina and Kuzyakov 2015; Hayat et al. 2017).

These exudates also correlate with a plant’s mode of photosynthesis. For example, C_3_ and C_4_ plants show variations in the types of exudates released into the rhizosphere. Dominant sugars in both types of plants differ, with the exudation of mannose, maltose, and ribose by C_3_ plants (Vranova et al. 2013). In C_4_ plants, inositol, erythritol, and ribitol are the dominant sugars exuded. C_4_ plants exude higher numbers of organic acids and amino acids compared to C_3_ plants. However, C_3_ plants exude more carbohydrates and organic carbons, (Nabais et al. 2011). C_4_ plant root exudates have varying pH. They are mineralized at different levels when compared to C_3_ plants (Tao et al. 2004).

Photosynthetically fixed carbon that is continuously secreted by plants as root exudates and low molecular weight antimicrobial compounds such as phytoanticipins and phytoalexins (Bamji and Corbitt 2017) appear to be a significant carbon cost for the plant. To cut down on the expended energy, plant exudation in the form of phytochemicals and rhizodeposits requires a high level of regulation. Recently, improvements have been made in decrypting the tightly regulated processes and stimuli alterations of root exudate flux demonstrating the complexity in the plant rhizosphere defense system. However, in the absence of pathogen induction, plants exude other high molecular weight compounds for defense (Alufasi et al. 2017; Preston 2017). The extent and nature of exudation varies with the age of the plant. Young plants exude carbon to the roots while older plants exude more carbon to the shoots. This increases root exudates in young plants compared to older plants (Pausch and Kuzyakov 2018; Pausch et al. 2013).

In one study, the diterpene rhizathalene A was found to be constitutively produced and released by non-infected *A. thaliana* roots (Vaughan et al. 2013). In this case, the diterpene was taken to be part of the regulated root defense system. Plants deficient in rhizathalene A have been identified as being more susceptible to insect herbivory. Thus, in addition to regulated root exudations, pathogen interactions can induce the synthesis, formation, and release of plant defense chemicals. Hence, the rhizobiome not only represents the site where pathogens encounter plants but is also a preventive microbial buffer zone serving as a line of defense that protects the plant against infection. Like the rhizathalene A, momilactone A is another antimicrobial diterpene that, in this case, is produced and secreted from the roots of rice (*O. sativa*) seedlings into the rhizobiome (Xuan et al. 2016).

Under laboratory conditions, various factors other than pathogen interactions can modify the root exudate composition, as observed in the accumulation of momilactone A in the leaves of blast fungus-infected rice plants (Prabakaran et al. 2017). Besides this ‘phytoalexin prototype’, the phytoanticipin (low molecular weight, antimicrobial compounds present in plants prior to any challenge by pathogenic microorganisms) level can also be upregulated by pathogen infection as observed in some labeling experiments which highlight the de novo synthesis and secretion of the compound t-cinnamic acid (Baetz and Martinoia 2014). Synergistic activities of momilactone A with other root exudates elicit activities of both phytoanticipins and pathogen-induced phytoalexins whose production and secretion are tightly regulated (Jabran 2017). On the other hand, a reduction of this defense compound is observed in the root hair tissues in the expression of ß-cryptogein which depicts the regulatory effect of this elicitor on the secretion of phenolic compounds into the rhizobiome (Baetz 2016; Jabran 2017). For example, the abnormal expression of the oomycetal elicitor ß-cryptogein in hairy roots of *Coleus blumei* mimics pathogen attack and, as a result, upregulates rosmarinic acid synthesis, which in turn displays antimicrobial activity (Bauer et al. 2015). Five antifungal phenylpropanoid root exudates were induced in *Hordeum vulgare* as a result of the interaction of the plant with *Fusarium graminearum* (Karre et al. 2017). With respect to microbe-microbe interactions, these exudates display antimicrobial activity and also interfere with quorum systems (QS) (Zúñiga et al. 2017). Large amounts of defense-related proteins are often released into the rhizobiome. This can be detected at the onset of flowering (Baetz and Martinoia 2014). It is believed that other antimicrobial compounds, volatile organic compounds (mVOCs), play important roles in long-distance interactions in the rhizobiome. In maize (*Zea mays*), benzoxazinoids form a class of defense molecules that are released during the emergence of lateral and crown roots (Guo et al. 2016). Although incompatible, their interactions do not affect the exudation of the monoterpene. Exudate secretion in the form of defensive compounds into the rhizobiome is strongly regulated by strategic processes that are controlled by various endogenous and exogenous stimuli. In agreement with this result, secretion of biotic stress-responsive proteins from the roots of *Arabidopsis* is also activated during compatible interactions (Baetz and Martinoia 2014). This shows the influence of microbial identity on a plant’s defensive root exudation.

Other than exogenous stimuli, endogenous evolutions also control variations in root exudate compositions. Specifically, the compatible interaction of pathogens or insects with *Arabidopsis* roots, but not mechanical wounding, induces the rapid secretion of 1,8-cineole, an antimicrobial agent (Wang et al. 2016). Similarly, plants utilize a strict defensive mechanism towards the latter phases of their life cycle. For example, this is observed in the increased production of antimicrobial phenolics in plant root exudates (Baetz and Martinoia 2014). In plant defense, terpenoids are significant contributors in terms of concentrations of above and belowground as part of root exudates (Rasmann and Turlings 2016; van Dam and Bouwmeester 2016). VOCs are released from plant roots as a direct defense mechanism (Ali et al. 2015); they have been described previously in their interactions with natural enemies of herbivores to provide indirect plant defense (Ali et al. 2015; Dorokhov et al. 2014). In contrast, an example of a direct belowground volatile defense compound is the already-mentioned *Arabidopsis* hairy root culture monoterpene 1,8-cineole whose secretion is induced by pathogen interaction (Mithöfer and Maffei 2016). In the same manner, secretion of pisatins from plant root tip tissues is also induced by pathogen interactions (Selim et al. 2017). Flavonoids constitute a large proportion of phenylpropanoid-derived secondary metabolites in plants, extending to root exudates. Isoflavonoid derivatives such as the phytoalexin pisatin from *P. sativum* are important antimicrobial compounds in legume plants (Jeandet et al. 2014). It has been suggested that nonvolatile and semi-volatile terpenoid phytochemicals can be secreted into the rhizobiome (Baetz 2016). Furthermore, activities of the diterpene hydrocarbon, rhizathalene A, have also been implicated as a component of mVOCs that is believed to be important in microbial community interactions in the rhizobiome (Sohrabi et al. 2017).

Acting in synergy, defensive root exudates form a diverse and flexible protective layer of chemical compounds in the rhizobiome. In addition to low-molecular-weight metabolites, high-molecular-weight root exudates also contribute to the immediate belowground resistance. For instance, belowground resistance was elicited by an *Arabidopsis* metabolite(s) towards root-feeding insects (Rasmann et al. 2017). Aside from the flavonoids, other highly bioactive exudates include tryptophan-derived metabolites such as the indole-derived camalexin and some glucosinolates (Khare et al. 2017). There has been the emergence of previously unrecognized proteins and DNA molecules in defensive activities. In line with these, overexpression of an *Arabidopsis* gene that regulates the biosynthesis of camalexin and salicylic acid (SA) confers resistance to nematodes in soybean (Youssef et al. 2013). Transcriptional activation of these genes as a result of infection activates the intrinsic synthesis, accumulation, and secretion of camalexin from the roots of *Arabidopsis*, while the disruption of these genes is characterized by low camalexin secretions and increased damage from pathogen attacks (Iven et al. 2012). Strigolactones, on the other hand, are involved in plant symbiosis with arbuscular mycorrhizal fungi during infection by root parasitic plants when released into the rhizobiome (Baetz and Martinoia 2014; Rasmann and Turlings 2016). The synthetic strigolactone analog GR24 inhibits the growth of an array of phytopathogenic fungi when present in the growth medium, indicating that rhizobiome-secreted strigolactones can alleviate some of the effects of pathogens through direct or indirect activities. One of these effects is carried out by interfering with the hormonal defense pathways, thereby contributing to explicit stress responses elicited by plants in the rhizobiome (Zhang et al. 2015).

Root VOCs can act as signaling molecules in attracting enemies of root-feeding herbivores and can also act as antimicrobial agents (Baetz and Martinoia 2014; Rasmann and Turlings 2016). The PsoR protein from rhizospheric pseudomonads has been found to be involved in the regulation of various antimicrobial-related genes that are efficient in biocontrol (González et al. 2013). They are henceforth regarded as a subfamily of LuxR proteins emanating from binding acyl-homoserine lactones (AHLs) response to signals (González et al. 2013). Solubilization of PsoR in the presence of macerated plants activates a Lux-box-containing promoter. This suggests that there is a plant molecule that binds to PsoR. Some genes involved in the inhibition and control of plant pathogens are a consequence of the activities of PsoR (González et al. 2013). Thus, production of 2,4-diacetylphloroglucinol depends on the presence and activities of PsoR.

In the plant-bacteria signaling cascade, it is important to identify the plant signal(s) regulating the communication system. A well-studied example of this class of metabolites is (E)-β-caryophyllene which is exuded by the maize root system in response to feeding by larvae of the western corn rootworm (Miller-Struttmann et al. 2013). Many plant-associated bacteria possess proteins similar to the quorum system protein LuxR. However, instead of binding to acyl-homoserine lactones, it alternatively binds to low-molecular-weight compounds (Baetz and Martinoia 2014). These activities have been studied in xanthomonads, rhizobia, and pseudomonads, demonstrating that this inter-kingdom signaling system is involved in regulating traits that are important for in planta colonization (Venturi and Fuqua 2013). Likewise, amino acid molecules such as canavanine can stimulate the functioning of one group of bacteria while suppressing the functioning of others (Cai et al. 2009).

### Regulation of root exudates

The connections between transport proteins, susceptibility to soil pathogens, and secretion of defense phytochemicals have not been extensively studied. In one of the few studies involving a multidrug and toxic compound extrusion (MATE) transporter in rice roots, the transporter was found to promote the exudation of phenolic compounds into the xylem (Baetz and Martinoia 2014). In another study, using *Arabidopsis*, it was reported that among the genes encoding ATP-binding cassette (ABC) transporter proteins, 25 out of the 129 genes in the genomic sequence of *Arabidopsis* (Garcia et al. 2004; Sánchez-Fernández et al. 2001), were involved in rhizosecretion which reflects their high expression level in root cells (Badri et al. 2008). Ultimately, members of both MATE and ABC transporters are capable of releasing root phytochemical aggregates into the rhizobiome. However, no MATE transporter has been reported to export root-derived antimicrobial compounds into the rhizobiome, thus questioning their potential to transport antipathogenic exudates and their general role in biocontrol. Similarly, silencing of the ABC transporter resulted in enhanced root sensitivity towards soil-borne pathogens, which can be attributed to the reduced secretion of antifungal compounds, such as the diterpene sclareol (Crouzet et al. 2013; Stukkens et al. 2005). The MATE transporter that has been analyzed to date appears to be connected with citrate release into the rhizobiome that confers aluminum resistance in plants. Furthermore, these studies revealed that consortia of ABC transporters may be used to trigger the release of a specific phytochemicals. ABC transporters are specific in their ability to moderate the export of several structurally and functionally unrelated substrates. However, besides the MATE and ABC transporters, there are a large number of yet to be characterized transporters, which might also be actively involved in belowground defense mechanisms. Traditionally, root exudation has been hypothesized to be a passive process largely mediated by diffusion, channels, and vesicle transport. Unlike the MATE and ABC transporters, the NpPDR1 transporters of *Nicotiana plumbaginifolia* are directly involved in plant defense against pathogen attack (Stukkens et al. 2005).

In future studies, it will be important to explore and elucidate the pathway regulation and modulations in transport protein mutants. This should improve our knowledge of the effects on root exudate patterns mediated by transport proteins involved in root exudation. In some studies on *Medicago truncatula* involving the ABC transporter and MtABCG10 gene, it was observed that the silencing of the transporter inhibits the synthesis of medicarpin in the phenylpropanoid pathway resulting in the possibility that isoflavonoid levels in the plant biotic stress response during phytoalexin de novo biosynthesis are being modulated by the MtABCG10 gene (Biala et al. 2013; Zhang et al. 2015). In another study, *Arabidopsis* mutant abcg30 was characterized by the secretion of lower molecular weight compounds into the rhizobiome while other mutant plants showed higher concentrations of defensive exudates in their rhizobiome (Badri et al. 2009). Invariably, ABC transporters modulate the synthesis and exudation of defense phytochemicals that can be modified by microbial elicitation (Mierziak et al. 2014). For example, the silencing of the MtABCG10 gene in *Medicago truncatula* results in an increase in the extent of root infection by *Fusarium oxysporum* (Hellsberg et al. 2015). It can be concluded from these findings that the AtABCG30 protein regulates transport systems, and the absence of this protein ultimately affects various metabolic processes. Recently, it was observed that nitrogen deficiency upregulates the biosynthesis of genistein (an isoflavone that binds to the NodD protein and initiates nodulation) from soybean roots (Jiao et al. 2017).

### Communication in the rhizobiome

Many rhizobacteria participate in quorum sensing (QS), the ability to detect and respond to microbial population densities, through the production and/or response to small molecule QS signals (García-Contreras et al. 2016). Rhizospheric cell-cell signaling is inevitable and many strains isolated from the rhizobiome have been reported to produce QS signals. The signals produced belong to a wide range of chemical classes, and one organism often combines multiple QS systems possessing different types of signals. Inter-kingdom signals have also been established with bacterial DSF (diffusible signal factor) signals which have been reported to elicit innate immunity in plants (Venturi and Keel 2016). Communications in the rhizosphere contain a regulatory response cascade complex that responds to a particular compound by eliciting the transcription of specific loci as a response. Many ascomycetes inhabiting the rhizobiome secrete signal molecules, mostly alcohols, that are active participants in specific plant developmental processes (Benocci et al. 2017). AHLs, a class of QS signaling molecule, can also act as inter-kingdom signals that regulate the expression of plant genes in the environment, induction of plant systemic resistance to stress, and as effectors of plant growth and development (Venturi and Keel 2016).

Some antibiotics synthesized by bacteria at low and non-inhibitory concentrations function as signaling molecules (Fajardo and Martínez 2008). Future work will need to determine the effects of antibiotics in rhizobiome communications and their role in shaping the microbiome. Unlike ascomycetes, many Gram-positive bacteria use pheromones instead of alcohols as signaling molecules (Yajima 2016); the feedback of these signals is likely to play modulatory roles both at the intra- and interspecies level. Rhizobiome communication between microbes largely determines the microbial community in the rhizobiome as well as influencing plant development.

VOCs can act as intra- and/or interspecies signals through the coordination of gene expression and influencing of biofilm formation, virulence, and stress tolerance (Altaf et al. 2017). There have been reports of some phytochemicals interfering with bacterial AHL-QS systems (Truchado et al. 2015). The first studied AHL-QS system is made up of a LuxI family synthase that synthesizes the AHL on interaction with the LuxR family regulator, thereby eliciting an increase in gene expression and ultimately altering the shape of the rhizobiome (Lareen et al. 2016). One of the most notable signaling networks is observed in legumes, which possess different nitrogen-fixing endosymbionts that act synergistically to form a stable inter-kingdom communication network, thereby efficiently aiding plant growth. The most common signals in gram-negative bacteria make use of AHLs. These signals can be important for the future engineering of the rhizobiome because, during a signal exchange between the host plant and the bacteria, various developmental mechanisms are activated. This highly developed and complex communication system among microbes plays a major role in harnessing and shaping the rhizobiome.

### Invaders keep off: rhizobiome in action

The rhizobiome is crucial to the growth, nutrition, and health of plants. It includes a great diversity of genomes from eukaryotes, viruses, and prokaryotes which are found in the plant ecosystem (Rout and Southworth 2013). All of these organisms form an ingrained interaction with the plant, and as a result, they aid in plant growth and may affect the health of the plant positively or negatively (Lapsansky et al. 2016). Positively, they can improve seed germination, seedling vigor, plant growth, nutrition, and plant development, while negatively they can cause diseases and stress competition for nutrients. The diversity of the rhizosphere microbial communities can directly influence host plants. According to Wagg et al. (2011), this richness in belowground diversity can help in maintaining plant productivity under adverse conditions. This is not due to the microbial community’s diversity but rather to its functional diversity, i.e., the different biochemical and physiological activities of the various microbes present in the soil.

**Fig. 1:**
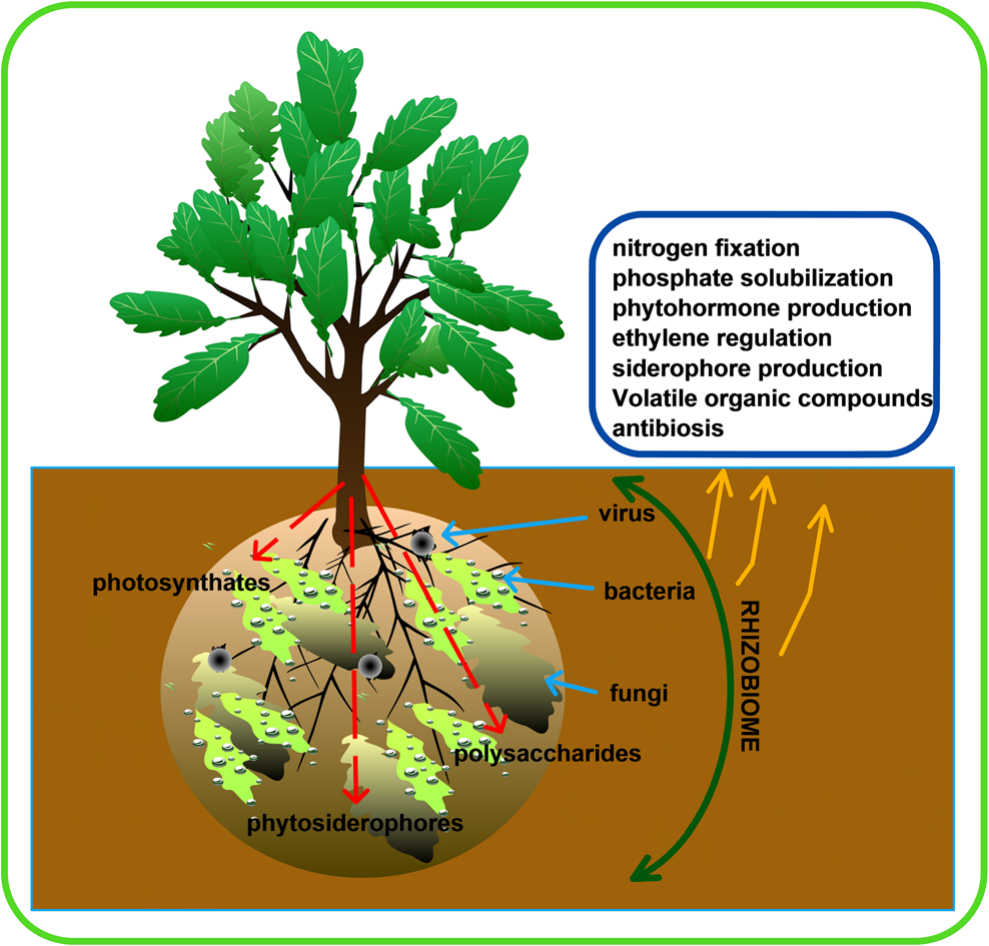
Rhizobiome diversity and effect on plant health. Photosynthates—products of photosynthesis in the form of simple sugars for energy. Functions majorly in energy production. Phytosiderophores—these enhance microbial activities in the soil. They relieve stress due to iron and zinc deficiencies through the acquisition of required iron and zinc for plant use. Polysaccharides—the most important form in plant is starch. It is a form of energy storage which is more complex than simple sugars

The positive effects of the rhizobiome on plants can be through the secretion of plant growth hormones, nutrient solubilization, pathogen antagonism, and plant immune system induction (Verbon and Liberman 2016). This establishment between rhizobiome and plants is influenced by the mutualistic interaction between the host plant and the surrounding soil (Chaparro et al. 2013). The deposition of fixed carbon and other exudates by plants to their surroundings causes an influx of microbes, ultimately increasing the microbial community diversity and microbial biomass due to the available nutrients (Fig. 1). The interaction of the rhizobiome with plant hosts is a gradual process that tends to be fully optimized as time goes on, eventually having a greater impact on plant growth and development (Bakker et al. 2012). Rhizobiome composition, multifariousity, and abundance vary due to many factors such as host plants, edaphic factors, and the microbial load. All of these factors effectively determine the survival of the host plant (Lakshmanan et al. 2014).

The rhizobiome impact on plant health is most evident in disease suppression as the competition for available nutrients is intense between beneficial and pathogenic microbes (Lareen et al. 2016). Most beneficial microbes need to increase in population so as to overcome the potentially deleterious effects of pathogens before they can invade plants, i.e., there is a need for beneficial microbes to be more numerous than the pathogenic microbes, leading to nutrient starvation for the pathogens, thereby rendering them ineffectual (Lareen et al. 2016). This phenomenon is referred to as “general disease suppression” and is attributed to the overall microbial activity (Berendsen et al. 2012). Thus, the diversity in the rhizobiome can be beneficial, mutual or non-beneficial, i.e., pathogenic (Anderson et al. 2010; Trdá et al. (2015).

### Beneficial microbes: a plant’s friends

Plant beneficial microbes (PBM) are important in the maintenance of plant health. They either aid in nutrient acquisition for the plants or help protect against pathogens. For example, in addition to the enhancement of nutrient availability for plants, arbuscular mycorrhizal fungi (AMF) and plant growth promoting bacteria (PGPB) can directly or indirectly influence a plant’s defense mechanisms (Di Benedetto et al. 2017).

Beneficial microbes in the rhizobiome aid in plant growth and development, as well as controlling pathogens using different mechanisms (Olanrewaju et al. 2017). Some of these mechanisms include biofertilization, bioremediation, and biocontrol (Babalola 2010; Olanrewaju et al. 2017). Examples of biofertilization activities of the microbes include nitrogen fixation, phosphate solubilization, and production of plant growth hormones, while biocontrol involves curtailing of the deleterious effects of plant pathogens through the synthesis of siderophores, regulation of ethylene levels, induced systemic resistance, and acquired systemic resistance, which are well documented (Fig. 1) (Glick 2015; Olanrewaju et al. 2017).

Rhizospheric microbes are very much involved in the uptake of trace elements like iron that exist primarily in an insoluble form, making them inaccessible to plants. This is where the use of siderophores comes to the forefront (Aznar and Dellagi 2015). By taking up these nutrients, they deprive the pathogens of access to these elements that leads to the inhibition of pathogen proliferation. Other notable mechanisms of biocontrol employed by beneficial microbes, in addition to the ones mentioned earlier, include quorum sensing interference, antibiosis, and competition for nutrients (Babalola 2010; Olanrewaju et al. 2017; Raaijmakers and Mazzola 2012). Most rhizobacteria and rhizospheric fungi also produce metabolites that inhibit the growth of pathogens (Ali et al. 2015; Saraf et al. 2014). In addition to bacteria, *Trichoderma* species also produce some antimicrobial metabolites (Mukherjee et al. 2012). Antibiotics can either act as growth inhibitors by inhibiting enzymes that are involved in cell wall biosynthesis, nucleic acid metabolism and repair, they can disrupt protein synthesis, they can also aid in the disruption of membrane structure, or be mediators of cellular signals, depending on their concentration. Finally, they also act against bacteria-biofilm formation and protozoa (Raaijmakers and Mazzola 2012).

Volatile organic compounds (VOCs) are other major metabolites produced by rhizosphere microbes. They are known to show plant growth promoting activities, and signals between host plants and the rhizobiome even though they are produced in small proportions compared to other metabolites (Ali et al. 2015). *B. cepacia*, *S. maltophilia*, *P. trivialis* and *P. fluorescens*, *S. plymuthica*, and *B. subtilis* are among the bacterial species that have been shown to produce VOCs (Ali et al. 2015; Saraf et al. 2014).

The plant immune system can also be triggered by some rhizospheric bacterial species, a system that is regulated in most cases by jasmonic acid and ethylene (Berendsen et al. 2012; Nambara 2013). During the interaction of plants with beneficial microbes, the jasmonic acid pathway may be activated, resulting in induced systemic resistance (ISR). During ISR, beneficial microbes activate the jasmonic acid-ethylene pathway which increases plant’s response time to infection by pathogens (Zamioudis and Pieterse 2012).

### Non-beneficial microbes

Non-beneficial microbes include mostly pathogenic fungi and viruses, as well as some classes of bacteria. They include *Pectobacterium atrosepticum*, *Pectobacterium carotovorum*, *Rhizoctonia solanacearum*, *Dickeya dadanthi*, *Dickeya solani*, and *Agrobacterium tumefaciens* (Mansfield et al. 2012). Some pathogenic viruses make use of nematodes and fungi as transport vehicles into the rhizosphere of plants (Rochon 2009). Most nematodes are free-living, while others can either be ectoparasites or endoparasites (Rasmann et al. 2012). The endoparasites can further be said to be migratory or sedentary depending on their location on the plant root. Nematodes’ sensory organs aid in their movement in search of nutrients (Rasmann et al. 2012). Some human pathogens have also been discovered to negatively affect plant growth (Mendes et al. 2013). Bacteria that cause human infections may be resident in the rhizobiome. Many human pathogenic bacteria (e.g., *Pseudomonas aeruginosa*, *Burkholderia cepacia*, *Salmonella enterica*) can be as highly competitive for nutrients as the resident rhizosphere microbes. They secrete metabolites which often allow them to fully proliferate on plant surfaces, colonize, and outcompete other microbes (Berg et al. 2005). Similar to beneficial microbes, some non-beneficial microbes induce the plant’s salicylic acid pathway instead of the jasmonic acid/ethylene pathway (Nambara 2013). Such organisms include the *P. syringae* virulence effector HopI1 and the biotrophic fungus *U. maydis*, which causes smut disease in maize (Tanaka et al. 2015).

### Plant-rhizobiome interactions: molecular machinery in action

The mechanisms involved in plant-microbe interactions are complex. This process involves various levels of communications between organisms, activation and inactivation of genes, induction and repression of responses to various signals, and various pathways elicited in responses. In this review, only some of the major interactions and responses occurring in the rhizosphere are considered. In-depth studies are available in the works of Catherine and Joel (2018), Yusuke et al. (2018), and Boller and Felix (2009).

In recent years, pattern recognition has emerged as an important process in plant immune responses. The presence of pattern recognition receptors (PRRs) makes it possible for plants to perceive different molecular signatures peculiar to various classes of microbes interacting with it. As previously discussed, these interactions might be beneficial or non-beneficial (pathogenic). Damage by non-beneficial microbes may induce plants’ self-signals referred to as endogenous elicitors, such as plant elicitor peptides (PEPs) (Lee et al. 2018; Ruiz et al. 2018), or damage-associated molecular patterns (DAMPs) (Cavaillon 2018). Plants are able to detect the presence of microbes through PRRs that bind to microbe-associated molecular patterns (MAMPs). Some rhizobacterial metabolites also serve as MAMPs. They induce a positive response by systemic defense priming (Wiesel et al. 2014). MAMPs including chitin and flagellin are likely to be more common in the rhizosphere than DAMPs (Poncini et al. 2017) as MAMPs are produced by bacteria in the rhizosphere while DAMPs are produced by plants. The PRR-MAMP binding activates the plant’s basal defense mechanisms to fight invading pathogens (Rosier et al. 2018). PRRs’ perception of MAMPs or DAMPs activates the plant’s immune response. However, this often tends to be ineffective against well-adapted microbial pathogens (Boller and Felix 2009) as they have devised their own kind of immunity to this system. The immune response triggers a signaling cascade that activates transcription factors, reactive oxygen species, reactive nitrogen species, and defense-related genes (Ipcho et al. 2016).

Various legume-rhizobia model systems have been used to uncover many symbiotic genetic and molecular determinants (McCormick 2018; Wood and Stinchcombe 2017). The release of flavonoids from legume roots activates rhizobia nod factor (NF) transcription, i.e., lipochitooligosaccharides (LCOS). These nod factors account for rhizobia-host specificity (Behm et al. 2014). In plants, the lysine receptor-like kinase family, also referred to as LysM, are the receptors for LCOS. These receptors bind and respond to MAMPs (Antolín-Llovera et al. 2012). The binding and response of legumes to these bacterial signals have been implicated in LCOS’ ability to promote plant growth and health (Rosier et al. 2018). The binding of LysM to the receptor-like kinase of the NF receptor in the legume root hair epidermis triggers sets of signaling events such as cytokinin accumulation and calcium spiking, which facilitates root hair curling, as well as the development of subsequent rhizobia infections (Gamas et al. 2017; Maillet et al. 2011). Studies of plant LysM receptor evolution, functions, and mode of action in immune responses and symbiosis have been well documented in the works of Antolín-Llovera et al. (2012), Gust et al. (2012); Zipfel and Oldroyd (2017), Cao et al. (2017), and others. The role of LCOS on the nodulation of various plants is well established (Arunachalam et al. 2018; Li et al. 2017; Zipfel and Oldroyd 2017). The ability of any growth promoting microbe to effect changes in root architecture is significant to plant health. LCOS are able to do this and by doing so enhance plant nutrition capacity as an increase of the number of root hairs facilitates more nutrient uptake. This was observed by Oláh et al. (2005) who reported that the number of lateral roots of *Medicago truncatula* was increased upon the application of LCOS. Likewise, increases in root length, root surface area, and root tip numbers were also observed in *A. thaliana* upon the application of *Bradyrhizobium japonicum-*LCOS (Khan et al. 2011). The action of the LCOS on the non-legume *A. thaliana* might result from the suppression of the FLS2-MAMP receptor-based immunity through the degradation of flagellin sensing 2 (FLS2) protein (Liang et al. 2013). The FLS2 proteins are found on the plasma membrane.

## Current research gaps and future developments

Reference genomes for most plants are not yet available, partly due to the difficulty and cost of sequencing plant genomes that are typically hundreds to thousands of times larger than bacterial genomes. Thus, not all of the genes controlling plant root exudates have as yet been characterized and localized in specific loci in plant genomes. Once the genomic sequences of more plants become available, it will be necessary to carefully analyze this data and identify many of the unknown genes using transcriptomics, proteomics and metabolomics. These approaches should provide new insights into plant signal production and specific responses to the rhizobiome. Genes responsible for the regulation of these signals can be mapped and targeted for regulation

On the other hand, both *Medicago* and *Arabidopsis* have been sequenced and their genomes are available. Despite these, the secretome structure and activation are still not well understood. Multi-omics application can be applied in the detection, characterization, and regulation of these secretions. Through these, the source of these secretions can be identified as well as the regulatory mechanisms involved in the secretion of the exudates. Gene expression levels can be regulated and controlled to favor plant breeding.

Root exudates can attract various microbes to the rhizosphere, as beneficial microbes are attracted, so are detrimental microbes. The presence of these detrimental microbes causes harm to plants so that continuing research should focus on eliminating these detrimental microbes. Once the exudate interacting with the microbes are determined through metabolomics studies, it may become possible to genetically modify root exudate secretion to make it less advantageous for pathogens to persist in the rhizosphere.

Finally, little is known about the biochemistry and principles of evolution determining plant-microbiota composition both above and below the ground. This emphasizes the need for experimental systems to investigate mechanisms of microbiota structure and function. In many instances, PBM might be inadvertently removed due to the application of chemical pesticides that select against them.

## Conclusion

Plants help to maintain a stable rhizobiome through the production of fixed carbon resources. PBM in turn aid the growth of plants through root modification, nutrient acquisition, and protection against pathogens, among other functions. Thus, there is an important role played by the rhizobiome in maintaining the plant’s health. The rhizobiome provides the full support needed by plants for optimum growth and development. It is therefore recommended that more work should be done on these rhizospheric microbes and their interactions with one another and with plant hosts, to discover how to make them more efficient for continuous crop production. Although this aspect of plant-microbe interactions is still relatively new, experiments covering mechanisms of plant secretions and functional validation of these secretions from plants should be a focus. Also, methodologies should be developed for researches into plant exudates and secretions. Knowledge from these experiments can help in identification and characterization of functional genes encoding the secretion of these exudates. These genes can then be targets for improved plant breeding. Due to the complexity of the multicellular processes in response to external factors, the task of unraveling the mechanisms and validation of secretions can be frustrating but in the nearest future, it will be an effort that is worth the task.
